# Does cement augmentation of the screws in angular stable plating for proximal humerus fractures influence the radiological outcome: a retrospective assessment

**DOI:** 10.1007/s00402-020-03362-1

**Published:** 2020-03-04

**Authors:** Dominik Knierzinger, Ulrich Crepaz-Eger, Clemens Hengg, Franz Kralinger

**Affiliations:** 1 Department of Trauma Surgery, Medical University of Innsbruck, Anichstraße 35, 6020 Innsbruck, Austria; 2 Department of Trauma Surgery, Wilhelminenspital Wien, Montleartstraße 37, 1160 Vienna, Austria

**Keywords:** Proximal humerus fracture, Angular stable plating, Cement augmentation, Screw-tip augmentation, Low bone mineral density, Open reduction internal fixation

## Abstract

**Background:**

Screw-tip augmentation in angular stable plating offers new possibilities for the treatment of complex proximal humerus fractures. This retrospective analysis was performed to evaluate the radiological outcome of proximal humerus fractures treated with angular stable plates and additional screw-tip cement augmentation in patients over the age of 60.

**Materials and methods:**

A retrospective single centre analysis was conducted from June 2013 to December 2016. The minimum follow-up time was set to 6 months after surgery. Anatomical reduction and fixation were evaluated in respect to reattached tuberosities to the head fragment and the adequate restoration of the calcar area not showing any valgus or varus malalignment. Complete fracture healing was determined 3 months after surgery. Any failures such as secondary displacement, primary screw perforation, intraarticular cement leakage and avascular necrosis of the humeral head with concomitant screw cut-out were assessed.

**Results:**

In total, 24 patients (21 females; 3 males) at a median age of 77.5 (62–96) years were included. Five 2-part, twelve 3-part and seven 4-part fractures were detected. The measured median BMD value of 23 patients was 78.4 mg/cm^3^ (38.8–136.9 mg/cm^3^). Anatomical reduction was achieved in 50% of the patients. In most cases, the *A* level screws and the *B*1 screw were augmented with bone cement by a median of 7 (5–9) head screws used. Postoperative varus displacement was not detected in any of the patients. One patient (4.2%) sustained an early secondary displacement. Intraarticular cement leakage was detected in 3 patients (2 head-split fractures). Avascular necrosis of the humeral head was observed in 4 patients (16.7%). Revision surgery was necessary in four cases, using hemiarthroplasty twice and reverse shoulder arthroplasty the other two times.

**Conclusion:**

Screw-tip augmentation in angular stable plating for proximal humerus fracture treatment showed a low secondary displacement rate of 4.2% in patients suffering from poor bone quality. Nevertheless, the occurrence of avascular necrosis of the humeral head with mainly severe fracture patterns observed in this study was higher compared to previously reported results in the literature. Cement augmentation in head-split fractures is not recommended, considering the high risk of an intraarticular cement leakage.

## Introduction

The incidence of proximal humerus Fractures (PHFs) increased over the last decades by more than 28% and surgery rates have risen by more than 10% in patients aged 65 years and older [1]. Obviously, the prevalence of reduced bone mineral density accompanied by an increasing number of falls reflect the main risk factors in a growing elderly population.

Despite various surgical treatment modalities of displaced or unstable PHFs there is no general consensus about the optimal surgical treatment in the elderly so far.

The development of angular stable plates offered new mechanical properties, especially for the surgical treatment of PHFs [2]. Thereby, an improvement of the fixation strength was intended to conduct early shoulder rehabilitation by still providing healing of the fracture. There are great achievements of angular stable plates in PHFs, however, the high complication rates of up to 40% must be considered. Most frequently, screw cut-out with concomitant varus collapse and avascular necrosis of the humeral head (AVN) was reported [2–7]. Anatomical reduction of the fracture, restoration of the calcar area and an accurate subcortical screw anchorage decrease such complications [8–10].

Due to the impaired bone quality of the proximal humerus in osteoporosis, sufficient screw anchorage could not always be achieved. The microarchitecture of the bone and the amount of trabecular and cortical bone substance have a direct impact on the mechanical stability [11–13]. Concerning this matter, strengthening of the bone–screw interface in poor bone quality by applying polymethyl methacrylate (PMMA) bone cement around the tip of the screws represents a novel and reliable opportunity to improve the implant anchorage. Previous biomechanical investigations of angular stable plates with additional screw augmentation at the proximal humerus already emphasised the advantage for implant purchase in low bone mineral density [11, 14, 15]. Existing clinical data are rare and inhomogeneous in its outcome [16–18].

The aim of the present study was to evaluate and analyse the radiological outcome of PHFs treated with angular stable plates and additional screw-tip augmentation in patients over the age of 60. At a single centre, pre-, intra- and postoperative radiographs were assessed, and any failures, such as screw cut-out/perforation, secondary fracture displacement (varus collapse), intraarticular cement leakage and AVN, were analysed.

## Materials and methods

A retrospective data analysis of all patients who underwent surgery due to PHFs at the Department of Trauma Surgery at the Medical University Hospital Innsbruck was conducted. All medical reports between June 2013 and December 2016 were analysed. In total, 49 consecutive patients, who suffered from a PHF treated with angular stable plates and additional cement augmentation of the screws, were detected. According to the inclusion and exclusion criteria, 24 out of 49 patients were enrolled. Fifteen of the excluded patients had an uncompleted follow-up less than 6 months and 10 patients participated in another clinical trial.

The local ethics review board of the Medical University Innsbruck approved the study (AN2016-0157 364/4.20).

### Inclusion criteria

All patients over the age of 60 years who suffered from an isolated unilateral PHF treated with open reduction and angular stable plate fixation and additional screw-tip augmentation were enrolled into this study. A completed minimal radiological follow-up of at least 6 months, including plane radiographs and an initial CT scan, was mandatory. All surgeries were indicated due to a greater displacement and the angulation of the fragments in different fracture patterns according Neer’s criteria [19].

### Data assessment

All of the 24 PHFs were classified by applying the HGLS Classification System introduced by Sukthankar and Hertel using the preoperative CT scans [20]. Various fracture displacement types were investigated at the preoperative CT scans in more detail, with regard to medial hinge displacement resulting in a potentially greater head shaft displacement, head inclination regarding to the shaft and any head-split components or calcar fragments. A greater head shaft displacement was defined as a displacement between the head fragment and the shaft greater than the diameter of the shaft.

**Fig. 1:**
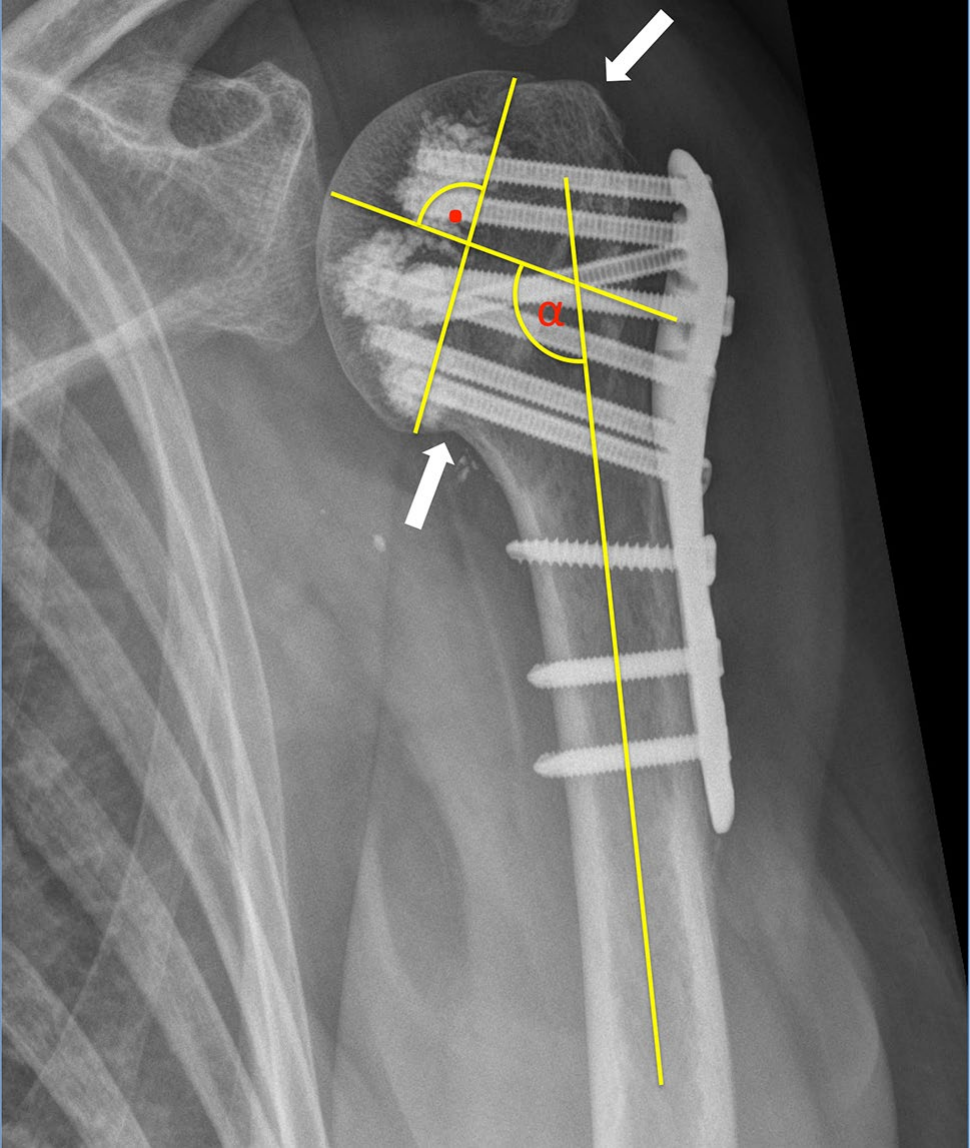
Showing the measurement technique to measure the head inclination, which was primarily introduced by Hertel et al. by measuring the α angle in a case with an accurate calcar restoration and adequate reattached tuberosity to the head fragment (each marked with an arrow) but with a varus mal-reduction (*α* = 120°) [21]

Preoperative plane radiographs (AP view, an outlet view and most often an axillary view), CT scans and intraoperative (true AP view, axillary view) as well as postoperative plane radiographs (AP, outlet and Velpau/axillary view) were assessed. An adequate anatomical reduction of the fracture was achieved when the tuberosities were reattached to the humeral head not showing any gap or displacement of more than 5 mm. Further, the head fragment must be reduced on the shaft resulting in an anatomical calcar restoration not showing any varus or valgus malalignment. Varus malalignment was defined as a head inclination of less than 125° regarding to the shaft. A head inclination of more than 145° was defined as a valgus malalignment. The surgical adjustment of the displaced fragments and the head shaft angle were assessed on plane AP radiographs by measuring the head shaft angle of the intraoperative radiographs and comparing them to postoperative radiographs when fracture healing was completed [21, 22] (Fig. 1).

Bony healing of the fracture was determined on plane radiographs 3 months after surgery.

The bone mineral density was measured on bilateral shoulder CT scans of the uninjured shoulder using a calibration phantom (European Forearm Phantom) [23].

**Fig. 2:**
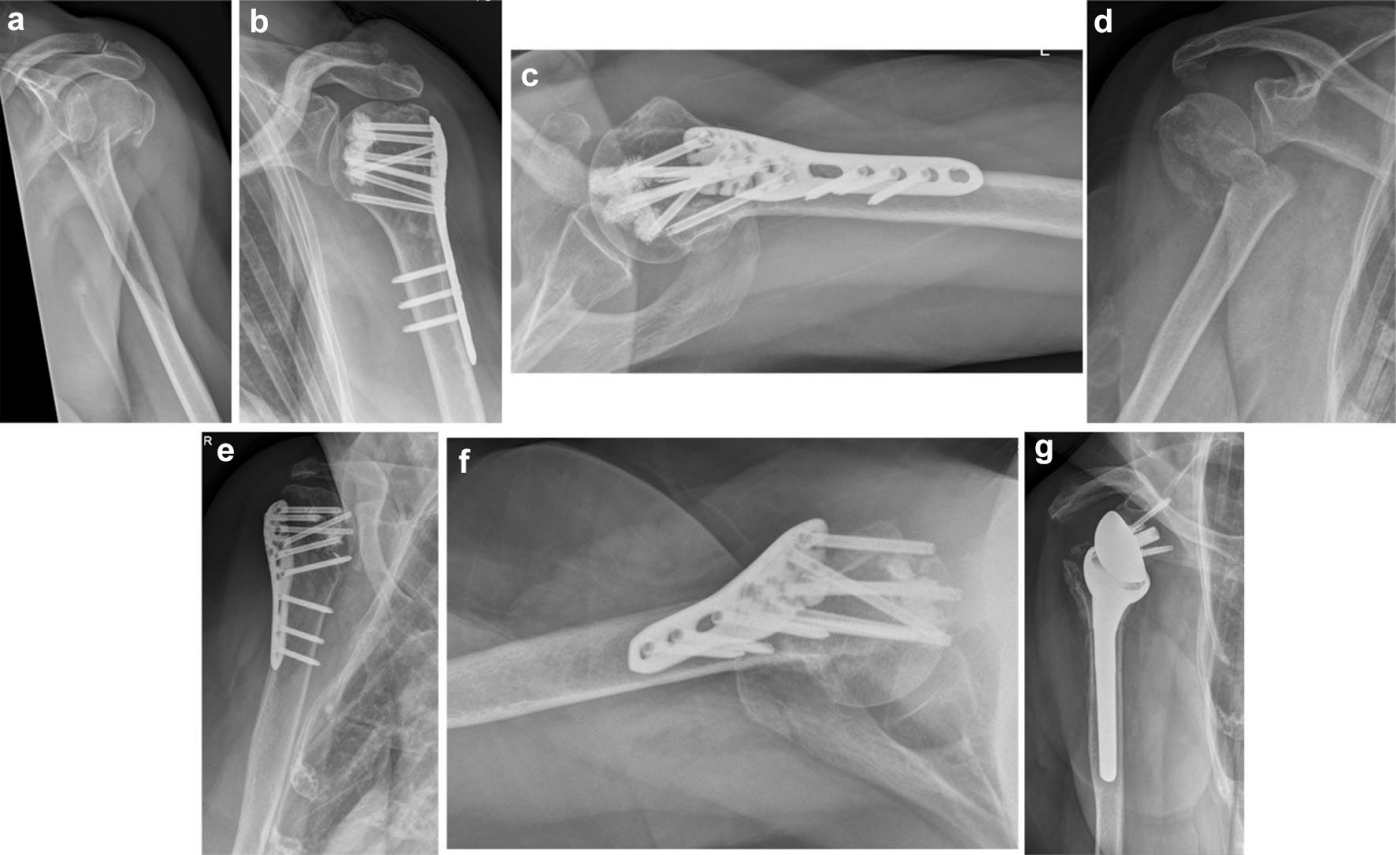
The first case **a**–**c** shows an accurately reduced PHF with an uneventful course. The second case **d**–**g** shows the only secondary fracture displacement (head fragment displaced) in this study with subsequent secondary screw perforation. Revision surgery with the application of reverse shoulder arthroplasty was necessary

Potential complications such as primary screw cut-out/perforation, secondary fracture displacement, intraarticular cement leakage and AVN were investigated. Primary screw perforation was defined as the tip of the screw crossing the border of the medial cortex of the humeral head. Secondary fracture displacement was defined as the reduced tuberosities being displaced > 5 mm and/or the inclination of the head fragment changing > 15° on the follow-up radiographs compared to the intraoperative ones. The appearance of an AVN was obvious when the humeral head lost its spherical shape on the radiographs. Intraarticular cement leakage was defined as surgery-related failure detected in the early postoperative radiographic check-up. Secondary screw perforation was assessed with the occurrence of AVN or secondary displacement of the head fragment (Fig. 2).

Primary screw perforation, secondary fracture displacement, intraarticular cement leakage and AVN were rated as positive, if one or more of these complications appeared in two different planes on the follow-up radiographs or on a CT scan, which patients obtained in case of a revision surgery.

### Surgical technique

Experienced trauma surgeons of a single trauma centre performed all the surgeries. Philos^®^ standard angular stable plates were used for all fractures (DePuy Synthes Companies, Zuchwil, Switzerland).

All surgeries were carried out via a deltopectoral approach. Fracture reduction and plate positioning were performed in a standardised fashion and controlled using the image intensifier. Cannulated and perforated screws were used for the humeral head; for the shaft non-cannulated angular stable screws were applied.

**Fig. 3:**
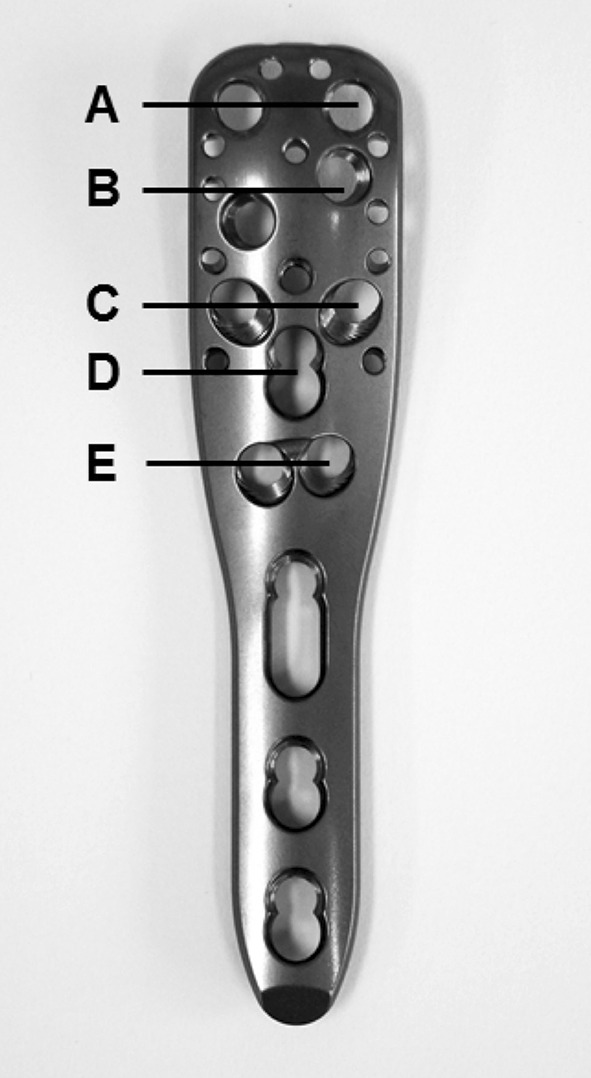
Different levels of a Philos^®^ plate. The anterior screw hole for each level was determined as 1 and the posterior screw hole was determined as 2, depending on whether it is the left or right arm

Cement augmentation was performed according the manufacturer’s guidelines (DePuy Synthes Companies, Zuchwil, Switzerland). Before cement application was conducted, the appearance of any intraarticular leakage was best possible excluded using a contrast agent (Jopamiro^®^, Bracco Imaging S.p.A., Milan, Italy) under direct fluoroscopy. The screws, which were determined as safe (no intraarticular leakage), were augmented with 0.5 ml cement (Traumacem V + ^®^, DePuy Synthes Companies, Zuchwil, Switzerland). The appropriate levels of the Philos plate regarding screw insertion and cement application on the humeral head were described in Fig. 3.

### Postoperative care

Postoperative immobilization was carried out with a shoulder-specific arm sling for 3 weeks. Pain-adjusted early passive and active assisted movements in full ROM were initiated 2 days after surgery.

## Statistical analysis

Descriptive statistics and frequencies were calculated using SPSS software version 24 (IBM Corporation, Armonk, NY, USA).

## Results

A total of 24 enrolled patients at a median age of 77.5 (62–96) years were analysed. Twenty-one were female and three were male. In 11 patients the right arm was affected, and in 13 patients it was the left arm. The median radiological follow-up was at least 12 (6–42) months. Five 2-part, twelve 3-part and seven 4-part fractures were surgically treated using an angular stable plate with additional cement augmentation of the screws. Three out of 24 fractures were head-split fractures, 6 fractures showed a calcar fragment and 7 fractures showed a displacement between the head and shaft fragment greater than the diameter of the shaft. Varus or valgus displacement was detected in 8 and 12 patients respectively and was adequately reduced in 6 (varus) and 11 (valgus) patients. The measured median BMD value of 23 patients accounted for 78.4 mg/cm^3^ (38.8–136.9 mg/cm^3^). The median medial hinge displacement amounted to 18.75 mm (0–42 mm) and the measured median metaphyseal head extension was 5.80 mm (0–24 mm). Anatomical reduction was achieved in 12 patients out of 24; medial hinge restoration was obtained in 15 patients out of 24 (62.5%). The median head shaft angle was intraoperative 130.5° (115°–139°) and did not increase or decrease compared to the cured fracture situation (129.5° [111°–137°]). Fracture healing was obtained in 20 patients out of 21 (95.2%) 3 months after surgery, excluding the 3 patients, who had to undergo an early revision surgery.

In total, a median of 7 (5–9) head screws of the angular stable plate were used for fracture fixation. Bone cement was applied at a median of 4 (2–7) screws.

One patient sustained an early secondary displacement (retrotorsion of the head fragment) 7 weeks after surgery. Three months after index surgery revision was indicated using reverse total shoulder arthroplasty. Further secondary displacements, especially varus-displacements, were not observed. Primary screw perforation with subsequent small amounts of intraarticular cement (not causing any severe erosion on the joint) was observed in three patients (2 of them had head-split fractures) at the postoperative radiographic follow-ups. At two patients the screw perforation was detected at the *C*-level and once at the *E*-level of the angular stable plate. Two of these three patients developed an AVN later on. In total four patients developed an AVN, two of them within 1 year and the other two after 2 years. Revision surgery was indicated in three patients, who developed an AVN, twice using hemiarthroplasty by anatomically healed tuberosities and once using reverse shoulder arthroplasty due to an extended rotator cuff lesion (Table 1).

**Table 1:** Summary of the occurred complications with a median follow-up period of 12 (6–42) months

Adverse events	Patients (total 24)
Secondary displacement	1 (4.2%)
Primary screw perforation → intraarticular cement leakage	3 (12.5%)
AVN	4 (16.7%)
Revision surgery	4 (16.7%)

In general, surgery was performed at a median of 3 (0–8) days after the injury occurred. None of the 24 patients suffered from a superficial or deep wound infection.

The main results were summarised in the table below (Table 2).

**Table 2:** Summary of the assessed data

Total number of patients	24
Gender	
Male	3
Female	21
Age	77.5 (62–96) years
Fracture pattern	
2 part	5
3 part	12
4 part	7
Fracture characteristics	
Head-split	3
Greater head shaft displacement	7
Calcar fragment	6
BMD mg/cm^3^	78.4 mg/cm^3^ (38.8–136.9 mg/cm^3^)
Medial hinge displacement	18.75 mm (0–42 mm)
Medial metaphyseal extension	5.80 mm (0–24 mm)
Fracture angulation	
Varus deformity	8
Valgus deformity	12
Postoperative head-shaft angle	130.5° (115°–139°)
Cured fracture head-shaft angle	129.5° (111°–137°)
Anatomical reduction	
Yes	12
No	12
Medial metaphyseal restoration	
Yes	15
No	7
Total head screws	7 (5–9)
Cemented head screws	4 (2–7)
Time from the initial trauma to surgery (days)	3 (0–8)

The values were presented as median, minimum and maximum values. Distances were measured in millimetres (mm) and angles in degrees (°)

### Screw augmentation

**Fig. 4:**
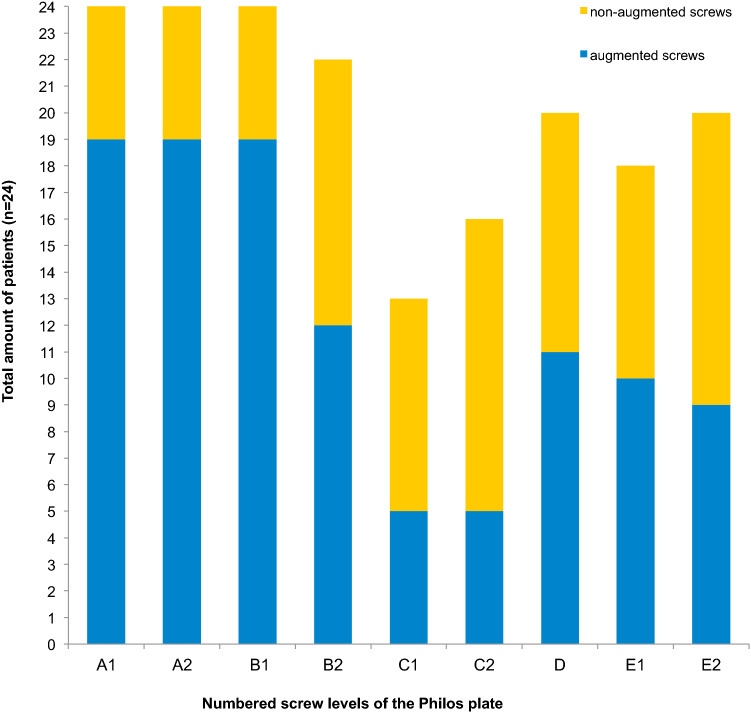
Amount of cemented versus non-cemented screws. Representing the distribution of cement augmentation at the different screw levels of the Philos^®^ plate. The blue columns represent the augmented screws per each screw hole and the orange columns the non-augmented screws

In 80% both screws at the *A* level and the *B*1 screw (convergent ones) were augmented. Augmentation at the *D* level was performed in 46%. The screws at the *E* level were augmented in half of the cases (Fig. 4).

## Discussion

Besides the occurrence of AVN, secondary screw cut-out with subsequent varus collapse of the head fragment in PHFs reflects the most common complication in angular stable plate fixation [2, 3, 5, 6, 9, 10, 24, 25].

The main findings of the present study, which included 24 highly selective patients at a median age of 77.5 (62–96) years with a median BMD value of 78.4 mg/cm^3^ (38.8–136.9 mg/cm^3^), are the fracture healing rate of 95.2% within 3 months, the low secondary displacement rate of 4.2%, an AVN rate of 16.7% and a total revision rate of 16.7%. Primary screw perforation accompanied by intraarticular cement leakage was observed in 12.5% of the patients.

Cement augmentation of the screws resulted in this study in a secondary displacement rate of 4.2% without any varus collapse compared to approximately 13.7–16% in conventional angular stable plating [2–4, 26, 27]. Regarding the low assessed anatomical reduction rate of 50% and the medial hinge restoration rate of 65%, screw-tip augmentation reduced the development of a secondary displacement in a highly selective patient population by enhancing the screw purchase in the subchondral area of the head. Due to this strengthening effect of cement augmentation, variances in anatomical reduction may be even more tolerated. However, we highly recommend striving for an appropriate anatomical reduction, particularly an anatomical medial hinge restoration in angular stable plating of PHFs. Even when applying additional screw-tip augmentation, the well-reported potential failure criteria have to be considered [10].

The total revision rate using screw-tip augmentation in this study accounted for 16.7% and is located in the lower range of comparable, previously published data of conventional angular stable plating in PHFs, which showed revision rates from 13% up to 28% in an elderly population [2, 4, 7, 16, 18].

A prospective, randomized, controlled multicentre study by Hengg et al. showed no benefit of screw augmentation in an underpowered study population of 65 patients in total, who completed the 1 year follow-up [16]. Mechanical failures (loss of reduction, humeral head impaction) occurred in totally 9 patients (13.8%; 5 in the augmentation group). An AVN was observed in 3 patients (10.3%) of the augmentation group and in 2 patients (5.6%) of the non-augmentation group. Overall, there was no significant difference in the occurrence of adverse events and the clinical outcome between both groups. In a recently published retrospective study by Siebenbürger et al., similar clinical and radiological results were shown compared to the multicentre study by Hengg et al. [18]. Katthagen et al. showed in their case–control series a reduced mechanical failure rate through a similar clinical outcome after cement augmentation compared to conventional plating for PHFs in the elderly [17]. All of these studies were focused on both the clinical and radiological outcome but did not evaluate the radiographs in detail compared to this study. In general, the radiologically detected complications are comparable to this study. No intraarticular cement leakage was reported in these studies except in the study by Hengg et al., who observed this adverse event in one patient [16]. In this study, primary screw perforation was observed twice at the *C*-level and once at the *E*-level, with subsequent intraarticular cement leakage without any clinical relevance. Therefore, no revision surgery was necessary in any patient due to this adverse event. A contrast agent was administered under fluoroscopic control before cement augmentation was performed at every potential screw. Nevertheless, different radiological views in various arm positions should be conducted and evaluated more carefully to exclude any primary screw perforation before applying bone cement [28]. Further, cement augmentation of the screws at the *C*-level (divergent configuration of the screws) should be avoided due to a higher risk of primary screw perforation with subsequent cement leakage. Screw-tip augmentation in head-split fractures cannot be recommended due to the high possibility of an intraarticular leakage in this fracture pattern (observed in 2 patients).

In general, cement augmentation of the screws was mainly performed at the upper screws of the plate (in 80% both *A* screws and the *B*1 screw). This reflects a recommendation of Unger et al., who gained significantly more load cycles in a biomechanical varus-bending test when performing cement augmentation of the *A* and *B* levels [11]. Screw augmentation at the *E* level was performed in half of the cases to gain more stability in the calcar area. Nevertheless, no additional biomechanical advantage in cement augmentation of the lower screws of the plate has been reported yet. According to histomorphometric assessments of the proximal humerus, the highest bone density is located in the subchondral area of the head and decreases towards the metaphyseal area [29]. A finite element analysis reflecting biomechanical testing showed the importance of an accurate screw length, not shorter than 8 mm, mostly influencing the calcar area to reduce cut-out failure and secondary varus collapse [13]. Therefore, it could be supposed that an increased stability in angular stable plating for PHFs can be achieved through accurate screw length at the calcar area with additional screw-tip augmentation in patients suffering from low BMD.

The AVN rate of 16.7% was higher than in previous reports (4–10%), except in the study by Jost et al. reporting an AVN rate of 30% in their patient collective [2–4, 7, 16, 18, 24, 26, 30]. The amount of cemented head screws did not show any difference in the 4 patients who suffered from AVN with a median of 4.5 (2–7) augmented screws compared to the 20 other patients with a median amount of 4 (2–7) augmented screws. It could be supposed that the PMMA cement administered into the cancellous bone of fracture areas in the proximal humerus may compromise the subchondral blood supply of the head. However, this stays in contrast to the findings of Goetzen et al. [31]. They reported no subchondral or cartilage lesions in an in vivo sheep model after subchondral PMMA bone cement application in the distal femur and proximal tibia. Obviously, this finding is not completely comparable to the proximal humerus, especially not for fracture cases, but it could be supposed, that screw-tip augmentation is not one of the main issues with regard to the development of an AVN. Even more severe fracture patterns in terms of a head-split component increase the occurrence of an AVN, which was observed in two of the four patients. Often, head-split fractures in low demanding elderly patients would be treated with shoulder arthroplasty [24, 25, 32]. Despite that these are borderline fractures, open reduction and angular stable plate fixation with additional cement augmentation present a reliable treatment modality. However, there is still no general consensus on a superior surgical treatment for these severe fracture patterns so far and the selection of an appropriate surgical procedure always depends on the philosophy, attitude and skills of the surgeon performing either joint preserving surgery or arthroplasty.

## Limitations

The retrospective design of this study presents some limitations.

Due to the small patient cohort of this study, concerning a highly selective elderly patient population, the results must be interpreted carefully and should be compared with other published articles regarding screw-tip augmentation in angular stable plating for PHFs. Common comparable clinical outcome scores were not available due to a lack of data. The slight variances of the arm position on various follow-up radiographs may lead to some inaccuracy in measurement [22].

The absence of a matched control group (age, BMD-values, fracture patterns) without additional cement augmentation of the screws in angular stable plating for PHFs would most likely highlight the mechanical advantage of screw augmentation. Any concerns in screw-tip augmentation for PHF treatment regarding the development of an AVN may be better ruled out when comparing it to a matched cohort without bone cement augmentation.

## Conclusion

Screw-tip augmentation in angular stable plating of PHFs using PMMA bone cement is a reliable surgical technique to enhance the implant bone anchorage, resulting in a low secondary displacement rate of 4.2% in patients suffering from osteoporosis. Compared to the existing literature, an increased occurrence of AVN’s was observed in mainly severe fracture patterns and must be taken into account. Any intraarticular cement leakage must be ruled out carefully by performing radiographs with an image intensifier in various arm positions during the administration of a contrast agent at the determined screws prior to cement application. Due to the high risk of an intraarticular cement leakage in head-split fractures, we do not recommend screw-tip augmentation.
